# Cost-effectiveness analysis of systematic fast-track transition from oncological treatment to specialised palliative care at home for patients and their caregivers: the DOMUS trial

**DOI:** 10.1186/s12904-020-00645-7

**Published:** 2020-09-15

**Authors:** Christine Marie Bækø Halling, Rasmus Trap Wolf, Per Sjøgren, Hans Von Der Maase, Helle Timm, Christoffer Johansen, Jakob Kjellberg

**Affiliations:** 1 VIVE – The Danish Center for Social Science Research, Herluf Trolles Gade 11, 1052 København K, Denmark; 2 Department of Oncology, Rigshospitalet, Copenhagen University Hospital, Copenhagen, Denmark; 3 Faculty of Health and Medical Sciences, Copenhagen University, Copenhagen, Denmark; 4 REHPA - The Danish Knowledge Centre for Rehabilitation and Palliative Care, Copenhagen, Denmark; 5 The Danish Cancer Society, Copenhagen, Denmark

**Keywords:** Cancer, Informal Care, Economic evaluation, Effectiveness, Utility, QALY, Accelerated, Palliative care, Psychological intervention

## Abstract

**Background:**

While hospitals remain the most common place of death in many western countries, specialised palliative care (SPC) at home is an alternative to improve the quality of life for patients with incurable cancer. We evaluated the cost-effectiveness of a systematic fast-track transition process from oncological treatment to SPC enriched with a psychological intervention at home for patients with incurable cancer and their caregivers.

**Methods:**

A full economic evaluation with a time horizon of six months was performed from a societal perspective within a randomised controlled trial, the DOMUS trial (Clinicaltrials.gov: NCT01885637). The primary outcome of the health economic analysis was a incremental cost-effectiveness ratio (ICER), which is obtained by comparing costs required per gain in Quality-Adjusted Life Years (QALY). The costs included primary and secondary healthcare costs, cost of intervention and informal care from caregivers. Public transfers were analysed in seperate analysis. QALYs were measured using EORTC QLQ-C30 for patients and SF-36 for caregivers. Bootstrap simulations were performed to obtain the ICER estimate.

**Results:**

In total, 321 patients (162 in intervention group, 159 in control group) and 235 caregivers (126 in intervention group, 109 in control group) completed the study. The intervention resulted in significantly higher QALYs for patients when compared to usual care (*p*-value = 0.026), while being more expensive as well. In the 6 months observation period, the average incremental cost of intervention compared to usual care was €2015 per patient (*p* value < 0.000). The mean incremental gain was 0.01678 QALY (*p*-value = 0.026). Thereby, the ICER was €118,292/QALY when adjusting for baseline costs and quality of life. For the caregivers, we found no significant differences in QALYs between the intervention and control group (*p-*value = 0.630). At a willingness to pay of €80,000 per QALY, the probability that the intervention is cost-effective lies at 15% in the base case scenario.

**Conclusion:**

This model of fast-track SPC enriched with a psychological intervention yields better QALYs than usual care with a large increase in costs.

**Trial registration:**

The trial was prospectively registered 25.6.2013. Clinicaltrials.gov Identifier: NCT01885637.

## Background

The vast majority of patients with advanced cancer prefers to spend their last period of life at home rather than in a hospital [1]. Nevertheless, hospitals remain the most common place of death in most western countries, where there is a trend towards hospitalisation of terminally ill patients [2–7]. For such patients, specialised palliative care (SPC) at home is an alternative that aims to improve the quality of life during end of life care. SPC at home an interdisciplinary collaboration between physicians, nurses, psychologists, social workers, and others to provide palliative care and symptomatic relief for patients and support of their relatives. Studies have indicated that SPC at home reduces the likelihood of cancer patients dying in hospitals and improves quality of life (QoL) [8–11]. Based on these studies, a randomised controlled trial (RCT), called DOMUS, aimed to investigate whether *an accelerated and coordinated transition from an oncological department to SPC at home* affected the time spent at home, symptom relief, QoL and the survival of patients with incurable cancer [12]. The accelerated and coordinated transition from an oncological department to SPC at home termed a systematic fast-track transition was supplemented with a dyadic psychological intervention. Psychologists are only involved in a limited number of SPC-teams. To ensure patients and closest relatives received similar treatment regardless of which SPC-team they were allocated to, a dyadic psychological intervention was attached to the intervention [12].

The DOMUS-study found no significant differences in time spent at home and survival time between the intervention and the control group [13]. In contrast, the intervention increased the QoL of the patients and reduced levels of anxiety and depression in the caregivers [13, 14].

SPC at home may also save healthcare-related costs, particularly costs associated with hospital admissions. However, the evidence from previous studies is inconclusive, to some extent explained by various SPC-models and patient groups included [15]. Reviews on SPC at home have indicated a need for structured economic evaluations of SPC-models [15–18]. For this reason, we aimed to investigate the economic consequences of the accelerated transition from oncological treatment at hospitals to SPC at home, as a secondary outcome of the DOMUS trial [12, 13].

This study’s objective was to evaluate the cost-effectiveness of a systematic fast-track transition from oncological treatment at hospitals to SPC at home supplemented with a dyadic psychological intervention, compared to usual care.

## Methods

### Design and setting

The DOMUS-study was a randomised controlled trial (Clinicaltrials.gov Identifier: NCT01885637) which involved patients with incurable cancer, admitted with the Department of Oncology at the Copenhagen University Hospital in Denmark. In total, 340 eligible patients, who provided the informed consent, were recruited in the study involving nine SPC-teams in the Capital region of Denmark in the span of 39 months between June 2013 and August 2016 [13]. More information about the inclusion criteria and definition of the population can be found in a protocol paper and the recently published original study on the DOMUS study [12, 13]. Patients were excluded if they were already in contact with a SPC-team, were unable to be discharged home, did not speak Danish, or were admitted at another hospital.

The recruited patients were randomly allocated 1:1 to two alternative arms: (a) the intervention arm – comprising a systematic fast-track transition from a comprehensive oncological centre to SPC at home combined with a dyadic psychological intervention, and (b) the control arm - comprising of usual care. Both groups were affiliated with the Oncological Centre and received oncological treatment if needed. In both groups, there was an option to include the closest relative (informal caregiver) determined by the patient. In total, 257 informal caregivers were included in the analysis. Thus, the dyadic psychological intervention was involving the patient and the informal caregiver and additional outcomes relating to the use by the close relatives of hospitals, primary care, public transfers, and the time used on informal care of the patients were measured.

### The intervention

The intervention comprised of a fast-track transition from oncological treatment at the hospital to SPC at home [13]. The treatment of the patients in the intervention arm included transferral from the hospital to their homes within a maximum of 5 days of randomisation. These patients were also referred to a SPC-team, who facilitated the palliative treatment at home in collaboration with the general practitioner and basic home care nurses. The dyadic psychological intervention was additionally provided to these patients and their closest relative. This psychological intervention was manualised i.e. a plan was set up before the patient was recruited. Two planned meetings between the psychologist, the patient and the informal caregiver were held within the first month, and subsequently the meetings were held whenever needed. In case of patient death during the trial period, three additional counselling sessions with the psychologist were provided to the informal caregiver - after 3 weeks, 5 weeks and 7 weeks of the patient’s death. More information about the psychological intervention can be found in the papers published by von Heymann-Horan et al. [14, 19, 20].

The intervention was discontinued 2 months after the patient’s death, but the follow-up investigation continued to up till 6 months after randomisation in the current economic evaluation.

### Usual care

Patients recruited in the usual care arm were discharged home as per the standard current practice; there was no accelerated discharge as performed in the intervention arm. The patients in the usual care arm could receive SPC and psychologist counselling, if these were required as per the standard treatment protocol, and if determined by the professional caregivers. This psychologist counselling was not manualised.

### Data

Despite the data used directly from the RCT (e.g. the surveys EORTC QLQ-C30 for patients and the SF-36 for the informal caregivers, and randomisation date) we have used Danish administrative register data. For each person participating in the DOMUS trial (identified with a personal identification number, CPR-number) information about their usage of public services was obtained 6 months before and 6 months after inclusion in the DOMUS trial. Data on health care usage, public transfers and socioeconomic characteristics were collected through register data from Statistics Denmark and National Health Authority. The following registers were used: The National Health Register, The Danish Psychiatric Register, The National Health Service Register for primary care, the Elder Indicators, DREAM database, and The Danish National Prescription Registry. For more information about the usage of these registers, see Additional file 1.

### Costs and utilities

The economic evaluation was a combined cost-utility analysis of four sub-analyses: (1) calculation of intervention costs, (2) calculation of cost of informal care, (3) register analysis of public and health service usage, and (4) estimating an effect using QALYs [21]. For evaluation of the costs, the hourly expenditure was estimated using the annual salary of an average publicly employed person within the relevant personal group; this information was collected by the office for municipal and regional wage data [22]. We assumed an effective working load of 1200 h a year, which is 52 weeks multiplied by 37 h minus private and public holidays, sickness leave, maternity leave, and working hours not directly related to patients (including section meetings, courses, conferences, breaks etc.).

#### Cost of intervention

The cost of intervention was concentrated around the psychologist counselling as the cost to SPC was included in the register analysis. The number of psychologist counselling and other contacts was manually registered by the project psychologists. The time required for each counselling session was collected by four of the project psychologists (see Table 1), and the costs for each session were determined by calculating the hourly expenditure, using the average salary of regional employed psychologists. In 2016, the average annual salary for a regional employed psychologist was €73,226, giving an hourly wage on €61 [22].

**Table 1:** Estimated time used for the psychological intervention

Components	Estimated time used
Start of intervention	10 min
Home conference	75 min
Counselling with patient	60 min
Counselling with caregiver	60 min
Counselling with patient and caregiver	90 min
Transport time to counselling or home conference	30 min
Telephone need assessment	25 min
Contact with specialised palliative teams	10 min
Writing in journals after counselling	15 min

Note: Transport time was assumed to 30 min, even though the project psychologists used 1 hr. The rationale behind this is that if implemented the psychologists would be affiliated with the local SPC-teams. Source: Four psychologists involved in the DOMUS-study

The calculation is based the costs if the intervention was implemented. A realistic transport time would be lower on average than that in the DOMUS trial as the project psychologists did not have access to a car and had to visit patients from the whole of the Capital Region. Therefore, transport time was assumed to be 30 min, even though the project psychologists used 1 hr on average. If a patient or a caregiver in the control group received psychologist counselling as per usual care, it would automatically appear in The Danish National Health Service Register since the counselling was based on referral from their general practitioner.

#### Cost of informal care

The costs involved in contribution of informal caregivers through caring for palliative patients are important in an economic analysis from the societal perspective [23–25]. We first estimated the total time spent on care of palliative patients by informal caregivers through the iMTA Valuation of Informal Care Questionnaire (iVICQ), which is a validated questionnaire-based survey to quantify the weekly time spent on care of the patient by informal caregivers [26, 27]. We assumed linearity between the observed data points and linear imputation to replace missing observations. Information of missing observations can be obtained by request.

After estimating the total time spent, we used the replacement cost approach for assigning monetary values for the same, through which the informal care was valued with the potential cost of replacing the informal caregivers’ time by professional assistance. The average annual gross salary of a municipal social and health worker (€51,361 in 2016) was used to derive the cost of informal care at €44.7 per hour. We chose the replacement cost approach over the opportunity cost approach, because most of the informal caregivers in this study were retirees, which implied that their opportunity cost can be hard to estimate [21, 23, 25].

#### Register analysis of public and health service usage

The Danish registers contain information regarding the public expenses to the health care sector (both primary and secondary), municipality health services (home care and home nursing services), and social transfers. It is important to note that the costs of the SPC-team are included in the expenses to hospitalization. Therefore, a trade-off between hospitalization costs and psychologist counselling cannot be determined. The health care costs due to public health insurance (the primary sector) include general practitioners, private medical specialists, physiotherapists, dentists, psychologists, and chiropodists. Most of the registers were accessible in the total period 2013–2016.

The evaluation of home care through the Elder Indicators was not straightforward and two assumptions regarding the data were needed. First, we only had access to this registry from January 2013 to June 2015, meaning that analyses done with information on home care was performed on a subset of the patients. Second, the delivery date of home care was unknown. The register merely included the month of delivery and the average weekly referred amount of home care in that particular month. We assumed that this average referred weekly time used on home care matched the actually utilized home care and was valid throughout the month (4.3 weeks in a month). Therefore, we have made two analyses. One including home care services and one excluding home care services. The results without home care services are seen as the main result.

Three types of home care activities were included, namely practical help, personal care and home care nursing. The valuation of *practical help* was estimated using the average annual gross salary of a municipal social and health personnel, which is €51,361 (€44.7 per hour) [22]. The valuation of *personal care* was estimated using the average annual gross salary of a municipal home assistant, which is €54,232 (€45.2 per hour) in the daytimes and €67,601 (€56.4 per hour) in the evening/night hours [22]. It was assumed that 60% of personal care takes place during daytime while the remaining 40% took place in the evening or night. The valuation of *home care nursing* was estimated using the average annual gross salary of a municipal home care nurse, which is €59,834 (€49.8 per hour). *Since the* register only provides information on the number of visits and not the time used, we assumed half an hour per visit.

#### Utility and cost-utility analysis

QALYs, which is calculated by multiplying a person’s life length by the value of the experienced QoL, is a measure of subjective health that assigns a value ranging from perfect health (1) to as bad as being dead (0) based on individual quality of life estimates [28].

To obtain QALY estimates, we used the disease-specific survey, the European Organisation for Research and Treatment of Cancer Quality of Life Questionnaire Core-30 (EORTC QLQ-C30), for patients, and a generic tool, the SF-36, for the informal caregivers. The questionnaires were administered at baseline, at 2, 4, and 8 weeks and after 6 months*.* For the caregivers, the questionnaires were additionally administered after approximately 2 weeks, 2 months, and 7 months of the patient’s death. The QALY was obtained by applying conversion algorithms and adjusted for baseline differences in QoL as described in previous studies [29–33]. The QALY estimates were calculated as the sum of the quality of life 6 months after admission with linear interpolation between the evaluation points [34]. Linear imputation was used with missing values.

Using the different costs and QALY estimates, we calculated incremental cost-effectiveness ratios (ICERs) to compare the costs of the two groups with the effect of the intervention in terms of costs per gained QALY, using the formula [21]:

$$ ICER=\frac{\Delta \mathrm{Cost}}{\Delta \mathrm{Effect}}=\frac{{\mathrm{Cost}}_{\mathrm{intervention}}-{\mathrm{Cost}}_{\mathrm{usual}}}{{\mathrm{QALY}}_{\mathrm{intervention}}- QAL{Y}_{usual}} $$

Based on the estimated ICER, it can be assessed whether the potential effect of DOMUS is worth the potential increased cost in a societal perspective. The societal perspective implies that the public transfers are not included in the ICER calculations. The focus is on the patients’ ICER value.

Throughout the cost-utility analysis, we have adjusted for missing values and adjusted for baseline in the QALY estimations.

### Statistical methods

All costs were discounted to 2016-prices using the Danish Consumer Price Index [35]. Costs data are usually not normally distributed; therefore, we bootstrapped costs to produce confidence intervals (CIs). Bootstrap simulations with 1000 replications were performed to estimate the ICERs. The results of the bootstrapped ICER were presented in cost-effectiveness planes. These showed differences in costs against differences in QALYs [21]. To obtain simultaneous estimates of costs and effect, we used an econometric method called SURE (Seemingly Unrelated Regression Estimator) [36, 37]. As the intervention and the control group might have different resource consumption at baseline, we have adjusted for baseline costs (cost of the previous 6 months). The adjustment is carried out with a regression-based method as descripted in van Asselt et al. 2009 [38]. Finally, we performed a sensitivity analysis to test the robustness of the ICER.

All data analysis and graphical illustrations were performed using SAS 9.3 and Stata 14.0. All statistical analyses were evaluated on a 5% significance level.

### Sensitivity analysis

To examine the sensitivity of the cost-effectiveness analysis a tornado diagram is conducted. A tornado diagram is a graphical illustration of univariate sensitivity analyses showing the influence of changing key assumptions on the estimated ICER, when other factors remain in their base values. The assumptions examined in this sensitivity analysis were: 1) the discount rate (halved or doubled), 2) all costs changed by −/+ 10%, 3) QALY in intervention group change by −/+ 10%, 4) the effective workload (1500 h vs. 900 h), 5) transportation time (15 min. vs. 60 min.), and 6) time used on psychological intervention changed by −/+ 10%. A vertical line in the tornado diagram represents the ICER from the main cost-effectiveness analysis to provide a reference to the changes in ICER. The main cost-effectiveness analysis is for patients only and includes costs of intervention in the period 2013–2016. A supplementary analysis including home care services is conducted for the period 2013 – June 2015. The horizontal bars in a tornado diagram represent the variation of the ICER given a change in key assumptions. The assumptions driving the outcome of the model are thereby displayed. The width of the bars indicates the uncertainty associated with each assumption.

## Results

### Baseline characteristics

Out of the 340 recruited patients, 14 dropped out, 4 were excluded (if they already had contact with a SPC-team, did not speak Danish or could not be discharged to home), and one was not in register data. Thus, 321 patients completed the study, out of which 162 patients were in the intervention group and 159 patients in the control group. Out of the 257 informal caregivers, 11 dropped out, 2 were excluded and 9 were not found in register data. Thus, 235 caregivers completed the study, out of which 126 were in the intervention group and 109 in the control group. The baseline characteristics indicate that patients and caregivers in both the groups were comparable in terms of most of the observed socio-demographic characteristics (Table 2). The only exception is the share of married/cohabiting among caregivers. Thus, randomization is considered successful and it is therefore not relevant to use regression models to correct for observable characteristics.

**Table 2:** Baseline characteristics of the patients and caregivers

Characteristic	Patients			Caregivers		
	Control (*N* = 159)	Intervention (*N* = 162)	***p*** value^**1**^	Control (*N* = 109)	Intervention (*N* = 126)	***p*** value^**1**^
Patient dead within six months	40%	43%	0.638	NA	NA	NA
Average age (years)	64	66	0.4551	61	60	0.3991
Employed^2^	24%	25%	0.746	46%	45%	0.923
Disability pension^2^	13%	9%	0.351	< 5%	5%	0.085
Elderly pension^2^	53%	57%	0.509	44%	38%	0.358
Women	51%	51%	0.998	65%	65%	0.993
Married/cohabiting	59%	65%	0.315	**84%**	**94%**	**0.022**
Average number of children at home	0.19	0.13	0.551	0.39	0.29	0.4421
Immigrant/Descendants	11%	5%	0.057	4%	6%	0.497
Education^3^:						
Basic school	22%	22%	0.965	21%	22%	0.920
High school	4%	6%	0.346	6%	3%	0.423
Short-cycle higher education	42%	42%	0.951	38%	39%	0.915
Medium-cycle higher education	26%	17%	0.086	25%	18%	0.177
Long-cycle higher and research education	6%	12%	0.061	10%	18%	0.077

Note: NA: Not applicable; ^1^*p* value of comparison between control group and intervention group. A *p* value marked in **bold** indicates that the difference between the two groups is significant on the 95% level. Based on Kruskal-Wallis test for age and number of children, otherwise based on t-tests; ^2^Defined by Statistics Denmark’s socioeconomics classification (SOCIO13); ^3^304 observations in intervention group and 227 in control group

### Costs

#### Cost of intervention

Out of 162 patients, we had information about the intervention costs for 159 patients. The estimated total cost of the psychological intervention over 6 months was €109,020, which was an average on €686 [CI €612 – €759] per patient corresponding to around 11 h [CI 10–12 h] per person. The costs of the SPC teams were included in the register analysis.

#### Cost of informal care

**Fig. 1:**
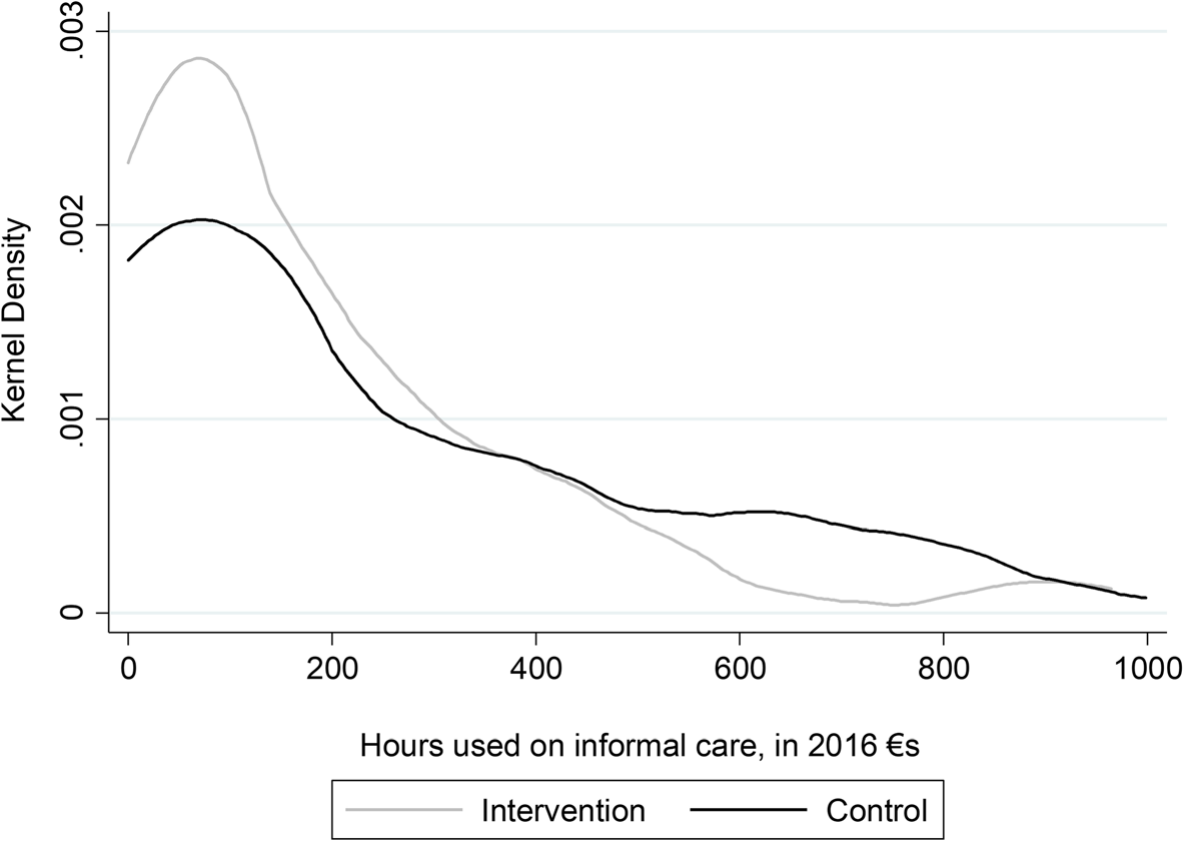
Kernel density curve of hours used on informal care within 6 months, in 2016€s. Note: A max of 1000 h is chosen to remove outliers from the illustration Source: DOMUS trial data

*Informal care* was estimated to cost around € 11,338 (95% CI 8680-13,996), corresponding to 253 h (95% CI 194–312) per caregiver in the intervention group, and € 12,052 (95% CI 9485–14,619), corresponding to 269 h (95% CI 212–326) per caregiver in the control group. The slightly lower costs and hours per caregiver in the intervention group was not statistically significant (*p* = 0.711). When using a Kernel density function curve [39] it was observed that the caregivers in the intervention group were using a lower number of hours on informal care, whereas the caregivers in the control group were using a higher number of hours on informal care (Fig. 1).

#### Register analysis of public and health service usage

The costs under this section include costs due to healthcare costs (Table 3) and public transfers (Table 4). For patients and caregivers in both the control and intervention groups, the highest costs were due to public transfers; however, the difference was not statistically significant (Table 4). The hospital costs were significantly higher for the patients and caregivers in the intervention group than the control group (€ 2726 vs. € 1366 for patients, *p* < 0.001; €282 vs. €100 for caregivers, *p* = 0.043). This difference was due to a larger number of contacts with the patients in the intervention group from the members of the SPC-team, when compared to the control group (1202 vs. 315 contacts over 6 months). Further, the average cost of each contact with members of SPC-team was significantly higher in the intervention group compared to the control group (€2852 vs. €2338, *p* = 0.008). The average costs in the intervention group (€2742) are calculated on all contacts with the hospitals and not only contacts with the SPC team. Thus, the average of all contacts can be lower than the average of contacts with SPC teams.

**Table 3:** Healthcare costs for patients and caregivers over 6-months, in 2016€s

**Patients**			
	**Control (N = 159)** **Mean (SD)**	**Intervention (N = 161)** **Mean (SD)**	***p*** value^**4**^
Hospitals^1^	**€1366 (2485)**	**€2727 (3208)**	**< 0.001**
Public health insurance^1,2^	€225 (303)	€214 (361)	0.779
**Total**^**1**^	**€1590 (2425)**	**€2941 (3267)**	**< 0.001**
Home care nursing^3^	€403 (996)	€578 (997)	0.270
Home care (personal care and practical help)^3^	€697 (1986)	€357 (1412)	0.209
**Total**^**3**^	**€2519 (3284)**	**€4132 (3870)**	**0.005**
**Caregivers**			
	**Control (N = 109)** **Mean (SD)**	**Intervention (N = 126)** **Mean (SD)**	***p*** **value**^**4**^
Hospitals^1^	**€100 (325)**	**€282 (869)**	**0.043**
Public health insurance^1,2^	**€164 (164)**	**€229 (303)**	**0.044**
**Total**^1^	**€264 (373)**	**€512 (932)**	**0.010**

Note: ^1^January 2013 to December 2016, *N* = 320 patients and *N* = 235 informal caregivers; ^2^This includes general practitioners, private medical specialists, physiotherapists, dentists, psychologists, and chiropodists; ^3^ January 2013 to June 2015, *N* = 160 patients, meaning that costs of hospitals and public health insurance are calculated for another period than the reported; ^4^*p* value marked in **bold** indicate that the difference between the two groups are significant on the 95% level

Source: DOMUS trial data and administrative data from Statistic Denmark and the National eHealth Authority

**Table 4:** Public transfers for patients and caregivers over 6-months, in 2016€s

Public transfers			
	Control (***N*** = 159) Mean (SD)	Intervention (***N*** = 161) Mean (SD)	***p*** value
Patients	€4666 (4624)	€4364 (4386)	0.548
Informal caregivers	€2066 (3279)	€2240 (3925)	0.715

Note: Public transfer are not included as costs in the ICER calculations. January 2013 to December 2016, *N* = 320 patients and *N* = 235 informal caregivers; p value marked in **bold** indicate that the difference between the two groups are significant on the 95% level

Source: DOMUS trial data and administrative data from Statistic Denmark and the National eHealth Authority

Hospital costs for caregivers appear as treatment not necessarily related to the patient’s disease. For the caregivers the costs due to public health insurance was significantly higher in the intervention group compared with the control group (€229 vs. €164, *p* = 0.044).

### Utility

**Fig. 2:**
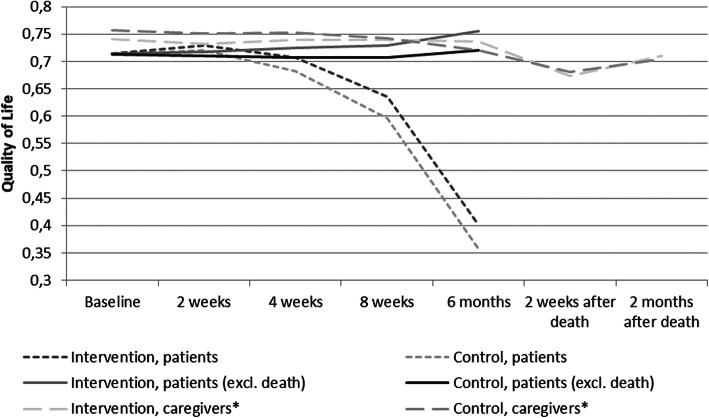
Utility scores on the EORTC QLQ-C30 for patients and SF-36 for caregivers. Note: Actual summary scores and the mapped utility scores are shown in Additional file 2. *2 weeks and 2 months after death is not necessarily after 6 months Source: DOMUS trial data. EORTC QLQ-C30 for patients and SF-36 for caregivers

Baseline utility scores for patients were similar between the intervention group and the control group (0.715 vs. 0.713; *p* = 0.887). Over the 6 months’ trial period, there was a reduction in QoL in patients belonging to both the groups (Fig. 2). The total QALYs gained for the patients over 6 months was significantly higher for the intervention group when compared to the control group (0.2612 vs. 0.2445; *p* = 0.026) when controlling for baseline QoL. The total QALY is the area under the curve in Fig. 2.

The reduction in the average QoL among patients is due to of the fact that patient death results in QoL score of zero. When the QoL of only the patients who survived for the entire duration of the study was considered, a rise in QoL was observed; this rise was slightly larger in the intervention group (0.379 vs. 0.365; *p* = 0.102).

The QoL among caregivers of patients belonging to both the groups were similar at baseline and throughout the study period. The lowest QoL recordings among the caregivers were observed at 2 weeks after the death of a patient, in both the groups. No significant differences in the mapped utility score between the intervention and control group were found for the caregivers (0.734 vs. 0.728, *p* = 0.630), why QALY estimates for caregivers are not used in the ICER calculations.

All actual summary scores and the mapped utility scores from QLQ-C30 (patients) and SF-36 (caregivers) at the different follow-up dates are presented in Additional file 2.

### Cost-utility analysis

The QALYs gained with the intervention were higher than by usual care. At the same time, the total costs, including costs of intervention and costs of home care services, were also higher for the intervention group. The ICER for patients was €118,292/QALY for the period 2013–2016. This is the base case and the main result. When the home care services costs were included and thus we look at the period 2013 – June 2015 the ICER for patients was €80,576/QALY.

**Fig. 3:**
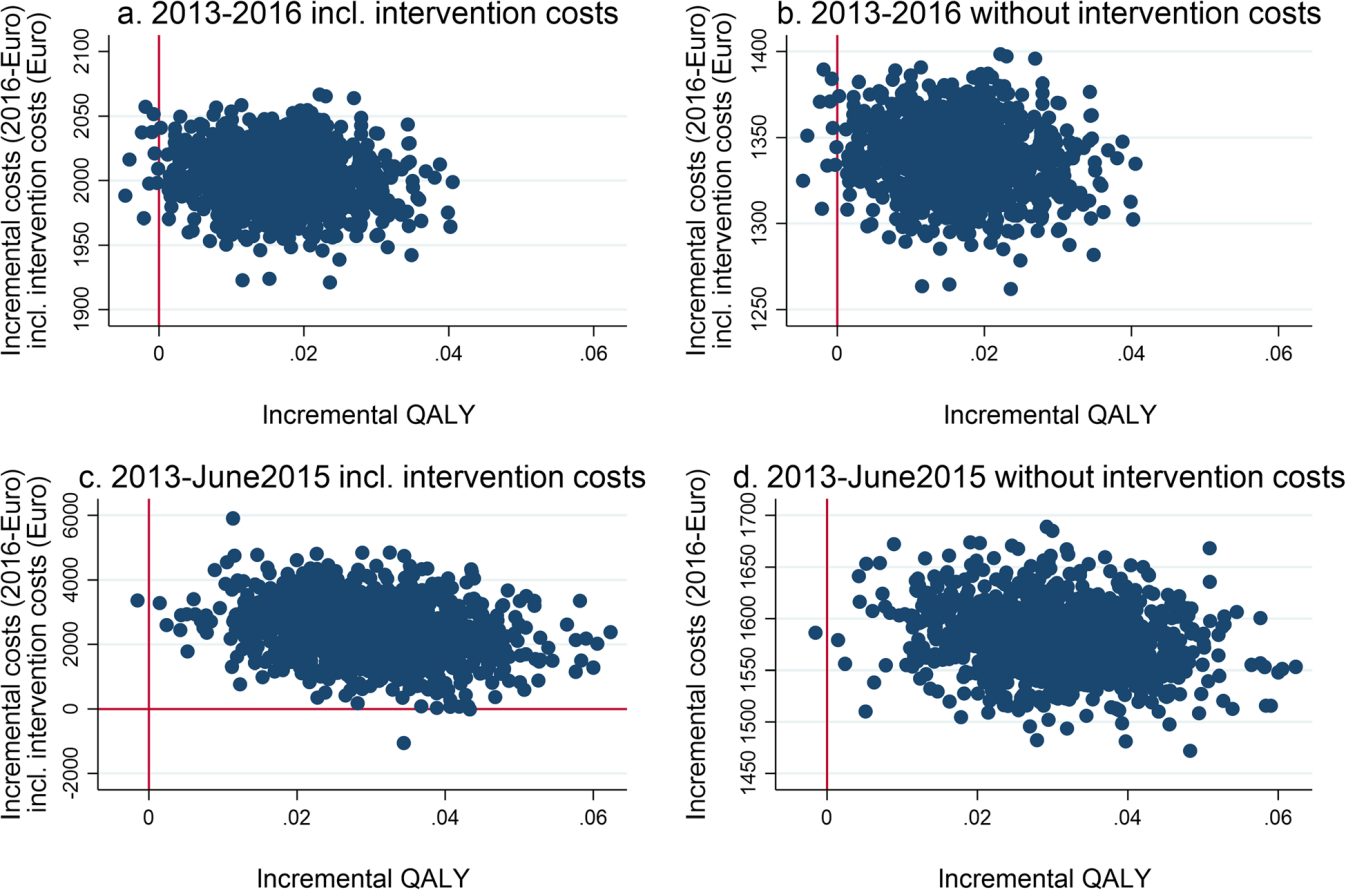
Cost-effectiveness planes of costs (health care costs, public transfers and costs of intervention) per QALY. Note: ICER: incremental cost-effectiveness ratio. QALY: quality adjusted life year. Based on 1000 bootstrap replications. **a** total costs 2013–2016 excluding home care services, **b** total costs 2013–2016 excluding home care services and cost of intervention, **c** total costs 2013 – June 2015, **d** total costs 2013 – June 2015 excluding cost of intervention

When costs of caregivers were also added the ICER was €131,185/QALY, and including home care services costs the ICER was €83,854/QALY. In the cost-effectiveness planes98.9 and 99.7%, respectively, (without and with home care services) of all replications occupied the north-east quadrant. This indicates that the intervention group had better health outcomes, but also higher costs than the control group (Fig. 3). The results are shown with and without the intervention costs, and for the two different periods.

**Fig. 4:**
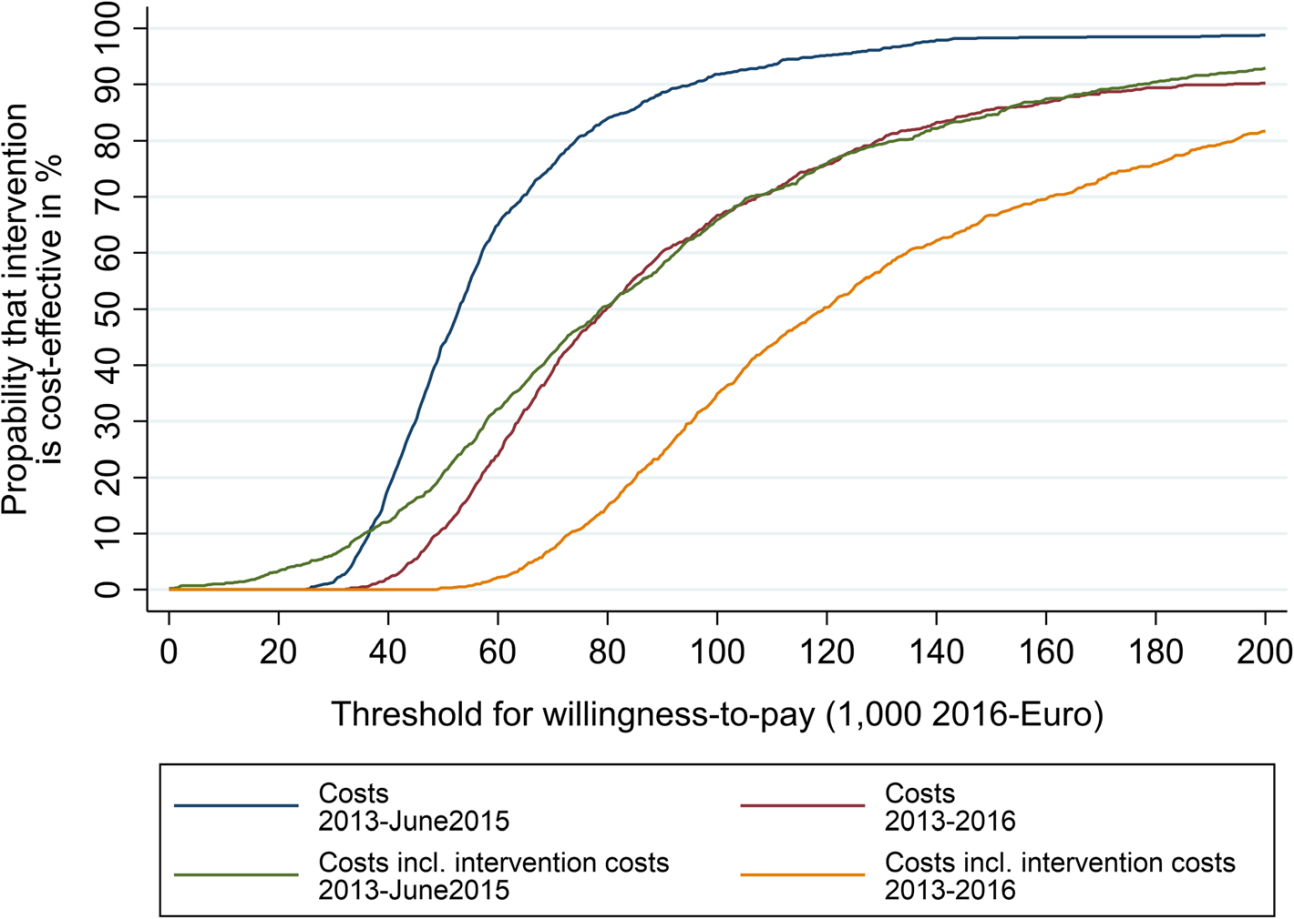
Cost-effectiveness acceptability curves for costs per QALY using different periods and with/without intervention costs. Note: The curves indicate the probability (y-axis) of DOMUS being cost-effective compared the usual care, given a specific threshold value (x-axis) for an additional QALY. Controlled for baseline utility Source: DOMUS trial data and administrative data from Statistic Denmark and the National eHealth Authority. EQORT for patients’ QALYs

The cost-effectiveness acceptability curves (CEAC) indicate that a threshold value of €78,800 and €119,200 per additional QALY (with and without home care services), which is associated with a 50% probability of the DOMUS intervention being cost-effective, when intervention costs is *not* included in the analysis. When intervention costs are not included in the analysis, a threshold value on €52,800 and €79,600 per additional QALY (with and without home care services) is associated with a 50% probability of the DOMUS intervention being cost-effective (Fig. 4). The variation in the threshold value derives from the two different periods of study, c.f. section 2.5 on register data.

### Sensitivity analysis

**Fig. 5:**
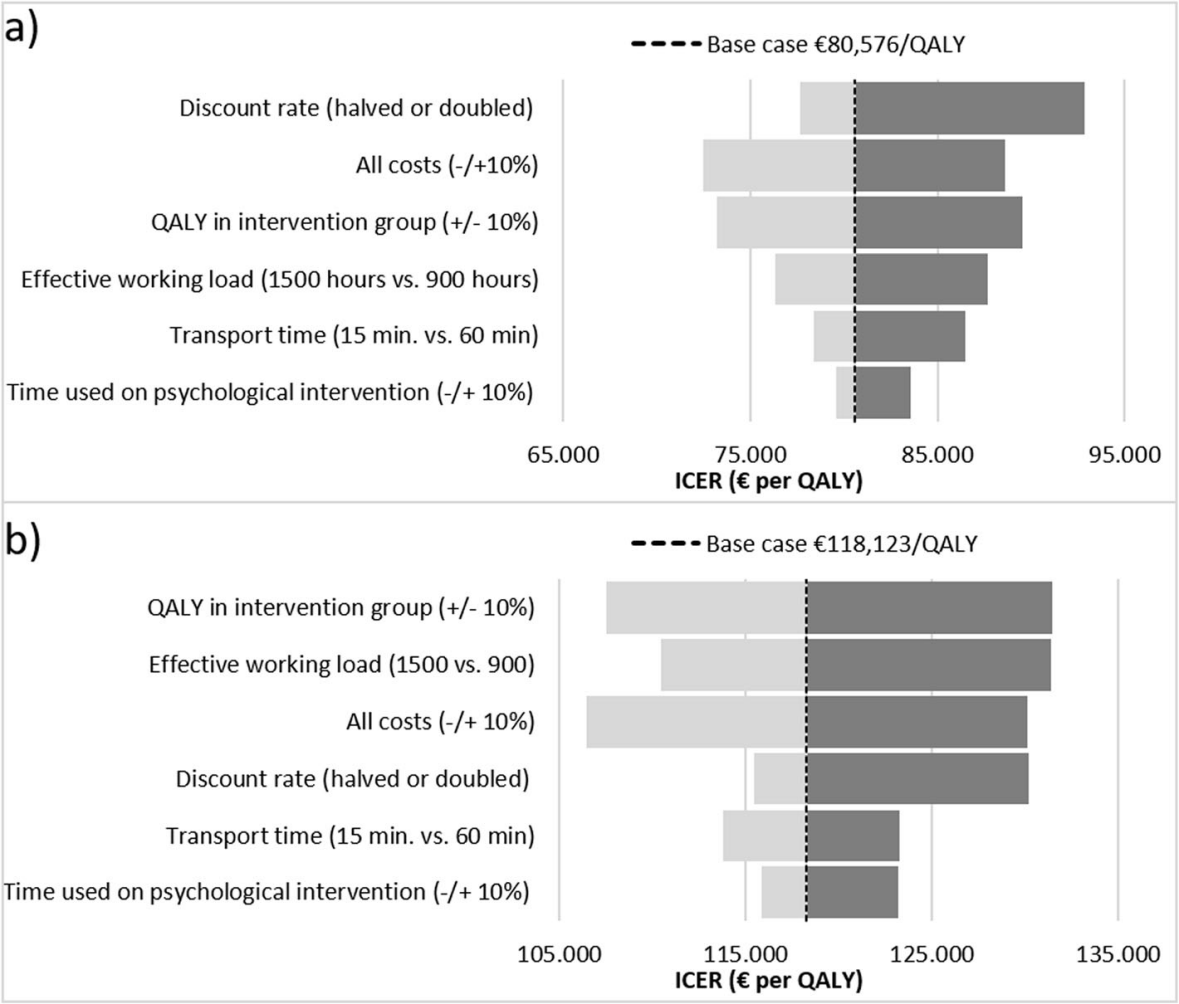
Tornado diagrams comparing the relative impact of key assumptions on the estimated ICER on €80,194/QALY. Note: In the period (**a**) 2013 – June 2015 and (**b**) 2013–2016. ICER: incremental cost-effectiveness ratio. QALY: quality adjusted life year. Costs include costs of intervention and costs of home nursing and home care. The vertical lines in the two tornado diagrams represent the ICER from the two main cost-effectiveness analyses (€80,194/QALY and €107,213/QALY) to provide a reference the changes in the ICER Source: DOMUS trial data and administrative data from Statistic Denmark and the National eHealth Authority. EQORT for patients’ QALYs

The sensitivity analysis revealed that the ICER varied from €72,518 to €92,872, when analyzing the period 2013 - June 2015 (Fig. 5). The variation is €90,723 to €146,155, when analyzing the entire period from 2013 to 2016 (Fig. 5). Largest impact was associated with a variation of 10% in all costs and the QALY estimate.

## Discussion

In the present study, a cost-utility analysis of the DOMUS fast-track intervention has shown that while the intervention improved the QALYs, it was also more expensive when compared to the usual care. The finding that most of the replications of QALYs were falling in the north-east quadrant of the cost-effectiveness plane, coupled with the analysis of the CEACs, indicate that fast-track intervention was not cost-effective compared to usual care, despite yielding better QALYs. In the current study, the lifespan was known for most of the patients, and it was not different between the two groups [13]. Thus, differences in the estimated QALY originated from potential differences in QoL*.* We found that the QALYs for the patients were higher in the intervention group than in the control group receiving usual care. The QALYs of the caregivers did not significantly differ between the groups. This corresponds to the previous literature. A meta-analysis from 2016 found that palliative care interventions were associated with improvements in patients’ quality of life and symptom burden, and finding for caregivers were inconsistent [40]. On the other hand, studies on the DOMUS trial found decreased anxiety and depression for caregiver [14, 20].

It is important to note that the results of this study are dependent on the protocol of the DOMUS trail, which means that the findings relate to this specific intervention. The evidence concerning cost-effectiveness from previous studies has been inconclusive as different SPC-models and patient groups has been investigated [15]. This study contradicts some findings of previous studies indicating that SPC at home reduces the likelihood of cancer patients dying in hospitals [8–11], which would reduce the hospital costs. This might be due to the specific definition of the SPC-model.

As mentioned QALYs were calculated using EORTC QLQ-C30 for patients and SF-36 for caregivers. EORTC QLQ-C30 was chosen for the patients, as it is one of the most commonly used measures of general health in cancer patients. Importantly, this measure has a great clinical utility describing the patients’ symptoms and disease [29]. Furthermore, it has been shown feasible to derive QALYs from EORTC QLQ-C30 [29]. Generic measures of health (e.g. EQ-5D and SF-36) can be used for cancer patients, but the evidence of validity and reliability is mixed, partly due to insensitivity for some medical conditions [29, 41, 42]. For caregivers the generic measure SF-36 was chosen, as it is possible to calculate QALYs from this. SF-36 gives information that is more detailed on the different domains than for instance EQ-5D, which could have been chosen as well.

Through the register analysis of costs, we observed that the intervention group had higher hospital costs over 6 months than the control group. This is in contrast to some previous literature finding that SPC does not result in changes in hospital admissions or even reduces hospital admission [15, 43, 44]. However, it is not a general conclusion from the literature [45]. As mentioned previously, the higher costs in this current study is due to a larger number of contacts with SPC-teams in the intervention group when compared to the control group. Likewise, the yearly report from the Danish Palliative Care Database in 2015 has indicated that the number of deaths in acute hospitals decreased and the number of deaths in SPC units increased when SPC-teams were involved [46]. These findings are contrasting studies indicating that patients with advanced cancer wishes to die at home [1].

Informal care is often particularly demanding with palliative patients, as the caregiver’s time spent on care is high. Even in the present study, the informal care formed a large chunk of the costs: we estimated the informal care to cost around €11,658 per caregiver corresponding to 261 h over a period of 6 months. It can be discussed whether this amount is accurate and whether it can be generalised to all caregivers of palliative patients. Despite that, the accelerated transferral from hospital care to home care in the intervention group would involve more demanding informal care for this group. However, contrary to our expectations, no significant difference in informal care was found between the intervention and control group. We found that the caregivers in the intervention group used a smaller number of hours on informal care, whereas the caregivers in the control group used more hours on informal care. Such a pattern might have happened because the accelerated transition to home may have generated patients, who are in need of a smaller amount of informal care from caregivers when compared to the patients in the control group due to their later transition to home. Another explanation could be that SPC supports the caregivers in receiving the appropriated help and had access to dyadic psychologists for a longer period.

There is no international consensus about the threshold value for cost-effectiveness in similar studies [47–51]. However, for appraisal of new interventions, a threshold in the range of around €10,000 to €40,000 has been used. The threshold is even higher in certain countries if other factors are important enough to outweigh the cost-effectiveness [50]. For example, thresholds at €80,000 have been used in cases of severe diseases in Norway [52]. We considered a threshold of €80,000 for our analysis since advanced cancer is a severe disease, and observed that the probability of the intervention being cost-effective was not high. The probability of DOMUS being cost-effective within the study period of study lies between 15 and 51% at a threshold of €80,000, when interventions costs are included. The uncertainty derives from the two different periods of study and whether the home care services are included.

The present study is the first full economic evaluation of a systematic fast-track transition from oncological treatment to SPC at home for patients with incurable cancer and their caregivers. A prominent strength of this study is that for the measurement of costs, a broad perspective has been used, since we included inpatient care, outpatient care, home care services, and public health insurance (including general practitioners, private medical specialists, physiotherapists, dentists, psychologists, and chiropodists) in the CUA analysis. We also analysed the public transfers and informal care. However, it should be kept in mind, that costs due to rehabilitation and nursing homes were not available and hence were not included. We also did not value the cost of potential production loss due to absence from work or decreased productivity, for both patients and caregivers, due to the advanced age of the patient and caregivers.

We fixed the time horizon for a limited period of 6 months, because our intention was that the last included patient should have the same “cost-frame” as the first included patient. Future research should examine costs on a longer time span. The number of patients with incomplete data may have introduced bias. Different assumptions were used to fill out missing data points. In addition, patients and caregivers in both groups may have potentially received psychological support and therapy from sources other than those which are publicly funded, e.g. privately financed or through workplace, patient organisations etc. Differences in the use of psychologists through these non-public channels have not been possible to include in the analysis.

## Conclusions

The DOMUS intervention included a systematic fast-track transition from oncological treatment to SPC enriched with a psychological intervention at home for patients with incurable cancer and their caregivers. We found that the quality of life for patients measured by the QALYs were higher in the intervention group compared to the control group receiving usual care. At the same time, the DOMUS intervention was also more expensive compared to usual care in a societal perspective. At a willingness to pay of €80,000 per QALY, the probability that the intervention is cost-effective lies at 15% in the base case scenario.

## Supplementary information

**Additional file 1.**

**Additional file 2.**

## Data Availability

The datasets generated during and analyzed during the current study are not publicly available as the individuals are identifiable.

## Abbreviations

CI: Confidence intervals
CEAC: Cost-effectiveness acceptability curve
CUA: Cost-utility analysis
DNPR: The Danish National Prescription Registry
DOMUS: The Danish Palliative Care Trial
DREAM: Danish Rational Economic Agents Model
EQORT QLQ-C30: European Organisation for Research and Treatment of Cancer Quality of Life Questionnaire Core-30
ICER: Incremental Cost-Effectiveness Ratio
iVICQ: iMTA Valuation of Informal Care Questionnaire
NHR: The National Health Register
NHSR: The National Health Service Register for primary care
PCRR: The Danish Psychiatric Central Research Register
QALY: Quality-Adjusted Life Years
RCT: Randomised controlled trial
SD: Standard deviation
SF-6D: Short-Form Six-Dimension Health Survey
SF-36: 36-Item Short Form Health Survey
SPC: Specialised palliative care
SURE: Seemingly Unrelated Regression Estimator
