# Supplementary material for: Cost-effectiveness analysis of systematic fast-track transition from oncological treatment to specialised palliative care at home for patients and their caregivers: the DOMUS trial
Source: BMC Palliat Care. 2020 Sep 15;19:142. doi: 10.1186/s12904-020-00645-7 (PMC7493170; doi:10.1186/s12904-020-00645-7)
Supplement: Supplementary file 1 — Additional file 1. [file 12904_2020_645_MOESM1_ESM.docx]

**Appendix A:** Applied registers

| Register | Description | Application in analysis 2013-2016 |
| --- | --- | --- |
| The National Health Register (NHR) | NHR contains information about inpatient and outpatient admissions at Danish somatic hospitals. This register includes information about diagnoses and the DRG-system | NHR was used in the cost analysis to quantify the utilisation of health care services in the secondary sector. Valuation was done with the DRG-system. |
| The Danish Psychiatric Central Research Register (PCRR) | PCRR contains information about inpatient and outpatient admissions at Danish *psychiatric* hospitals. | PCRR was used in the cost analysis to quantify the utilisation of psychiatric hospitals. Valuation was done with the DRG-system. |
| The National Health Service Register for primary care (NHSR) | NHSR contains information from health contractors in primary health care. This includes general practitioners, private medical specialists, physiotherapists, dentists, psychologists, and chiropodists. | NHSR was used in the cost analysis to quantify the utilisation of health care services in the primary sector. Valuation was done with public fees. |
| Elder Indicators | Elder Indicators is a register containing information about people referred to home care through their municipality (both at home and in nursing homes). Home care includes personal care, practical help, and home nursing. The register also contains information about the average referral hours of home care per week for people at home. | We had access to these data from 2013 to June 2015. The information about home care was used in the cost analysis. For people in nursing homes the hours per week was assumed to be the same as the average for people at home. See more in section 2.5. |
| Danish Rational Economic Agents Model (DREAM database) | DREAM contains weekly information on more than a 100 different public transfer payment (as sick leave and unemployment) | The information in DREAM was used in the cost analysis to determine public transfer payments. The valuation was based on a socioeconomic model from Denmark called SØM. |
| The Danish National Prescription Registry (DNPR) | DNPR contains information of dispensed prescription drugs, including price and type of drug. | DNPR was used in an aggregated analysis of the utilisation of prescription drugs before and after admission. When current study was conducted we did not have access to DNPR on individual level leading to the need of an aggregated analysis conducted by Statistics Denmark. |
| Other registers from Statistics Denmark | Information on age, gender, place of residence, marital status, socioeconomic classification, immigrant status, highest completed education | Used in the descriptive analysis |

Source: Statistic Denmark and the National eHealth Authority
