# Supplementary material for: Cost-effectiveness analysis of systematic fast-track transition from oncological treatment to specialised palliative care at home for patients and their caregivers: the DOMUS trial
Source: BMC Palliat Care. 2020 Sep 15;19:142. doi: 10.1186/s12904-020-00645-7 (PMC7493170; doi:10.1186/s12904-020-00645-7)
Supplement: Supplementary file 2 — Additional file 2. [file 12904_2020_645_MOESM2_ESM.docx]

**Appendix B:** Actual summary scores (QoL) and the mapped utility score from QLQ-C30 (patients) and SF-36 (caregivers)

| **Patients** | | | |
| --- | --- | --- | --- |
|  | **Intervention group** | **Control group** | ***p* value^4^** |
| Baseline | 0,715 | 0,713 | 0,887 |
| 2 weeks | 0,729 | 0,720 | 0,666 |
| 4 weeks | 0,707 | 0,682 | 0,380 |
| 8 weeks | 0,635 | 0,597 | 0,319 |
| 6 months | 0,402 | 0,356 | 0,317 |
| Mapped utility score* | 0,522 | 0,489 | 0,026 |
| **Caregivers** | | | |
|  | **Intervention group** | **Control group** | ***p* value^4^** |
| At baseline | 0,741 | 0,757 | 0,400 |
| At 2 weeks | 0,732 | 0,750 | 0,337 |
| At 4 weeks | 0,740 | 0,753 | 0,525 |
| At 8 weeks | 0,740 | 0,742 | 0,921 |
| At 6 months | 0,736 | 0,720 | 0,485 |
| 2 weeks after death | 0,673 | 0,681 | 0,700 |
| 2 months after death | 0,710 | 0,704 | 0,777 |
| Mapped utility score* | 0,728 | 0,734 | 0,630 |

*Adjusted for baseline differences and with linear imputation
